# 

**Published:** 1918-11-16

**Authors:** 


**ORDER THE PAPER.**

**In consequence of the No Returns Order of the Government, readers of THE HOSPITAL are requested to ensure a regular supply of the paper by placing an order with their newsagent or bookstall without fail.**

### Hammersmith Dispensary Staff.

Hammersmith Borough Council has now fixed its staff at the tuberculosis dispensary as follows:—Tuberculosis officer, salary £500 per annum, Mr. Robert Govan, M.D., M.B., Ch.B., M.A., age thirty-two, at present medical superintendent of the Vale of Clwyd Sanatorium, North Wales, ex R.A.M.C. officer, and previously tuberculosis officer of the Borough of Bermondsey. Two nurses, salary in each case £100 per annum, rising by annual increments of £10 to £130, plus war bonus: Miss A. S. Digby, certificated nurse at present engaged at the Birmingham Municipal Anti-Tuberculosis Centre, and previously at the Brompton Hospital, and Miss Zoë Langstaff, certificated nurse, with six years' sanatorium experience, and at present on the staff of the Paddington Tuberculosis Dispensary. Miss N. A. Rees, dispenser at the Fulham Dispensary for the Prevention of Consumption, has been appointed dispenser and clerk at a salary of £100 per annum, plus war bonus.

### Gifts and Bequests.

COLONEL H. S. BATES, of Alresford, Hants, has left £200 to Westminster Hospital.

MR. FRANK WILLS, of Eastbourne, left £1,000 to the North Cambridgeshire Hospital, Wisbech.

MR. J. W. PAYNTER, of Anglesey, has left £1,000 to the Anglesey District Nursing Association.

THE Notts Miners' Council has given £500 each to the Mansfield and Nottingham General Hospitals.

THE Chelsea Hospital for Women has received a donation of £10 10s. from the Pewterers' Company.

MR. ALFRED BAX, barrister-at-law, of Cavendish Square, bequeathed £1,000 to the Royal Hospital for Incurables.

THE Treasurer of the Royal Hospital and Home for Incurables, Putney Heath, has received five £1 Treasury notes from "C. M."

MR. HENRY BOWRING, of Torquay, bequeathed £500 to the Royal Merchant Seamen's Orphanage and £250 to the Mortenhampstead Cottage Hospital.

THE directors of the British and Foreign Sailors' Society have voted £3,000 in aid of the Malta Hospital Fund, for which an appeal has recently been issued by Lord Methuen.

THE Hon. Sir Thomas Mackenzie, High Commissioner of New Zealand, has received £50,000 for the Red Cross Fund from the Auckland Centre of the New Zealand Branch of the British Red Cross Society.

MR. JOHN INCH, of Hove, left the whole of the residue of his estate, after the death of his wife, between the Royal Caledonian Asylum, the Royal Masonic Institution for Boys, and the Royal Masonic Institution for Girls.

